# Supplementary material for: N-type and P-type series integrated hydrogel thermoelectric cells for low-grade heat harvesting
Source: Nat Commun. 2024 Oct 28;15:9305. doi: 10.1038/s41467-024-53660-0 (PMC11519491; doi:10.1038/s41467-024-53660-0)
Supplement: Supplementary file 1 — Supplementary Information [file 41467_2024_53660_MOESM1_ESM.pdf]

Supplementary Information for

N-type and P-type series integrated hydrogel thermoelectric cells for low-grade heat harvesting

Jiafu Shen[1], Xi Huang[1], Yu Dai[1], Xiaojin Zhang[1,*], Fan Xia[1,*]

[1]State Key Laboratory of Biogeology and Environmental Geology, Engineering Research Center of Nano-Geomaterials of Ministry of Education, Faculty of Materials Science and Chemistry, China University of Geosciences, Wuhan 430074, China

* e-mail: zhangxj@cug.edu.cn; xiafan@cug.edu.cn

Supplementary Note 1

The Seebeck coefficient (S) of thermocells is defined as:

$$S = \frac{\Delta E}{\Delta T} = \frac{\Delta S}{nF} \tag{1}$$

where, ΔE is the open-circuit voltage, n is the number of electrons in the redox reaction, F is the Faraday constant and ΔS is the entropy difference.

Based on the Nernst equation, if a redox reaction A + ne ↔ B reaches equilibrium, the equilibrium potential (E) can be expressed as:

$$E = E^{\ominus} + \frac{RT}{nF}\ln\frac{\alpha_{\mathrm{A}}}{\alpha_{\mathrm{B}}} \tag{2}$$

where, $E^{\ominus}$ is the standard potential, R is the gas constant, α_{A} and α_{B} are the activities of the oxidation and reduction species, respectively. According to the equation $\alpha = c\cdot\gamma$, where γ is the activity coefficient and c is the concentration, equation (2) can be expressed as:

$$E = E^{\ominus} + \frac{RT}{nF}\left[\ln\frac{\gamma_{\mathrm{A}}}{\gamma_{\mathrm{B}}} + \ln\frac{c_{\mathrm{A}}}{c_{\mathrm{B}}}\right] = E_{\mathrm{f}} + \frac{RT}{nF}\ln\frac{c_{\mathrm{A}}}{c_{\mathrm{B}}} \tag{3}$$

where, E_{f} is the formal potential.

$$E_{\mathrm{f}} = E^{\ominus} + \frac{RT}{nF}\ln\frac{\gamma_{\mathrm{A}}}{\gamma_{\mathrm{B}}} \tag{4}$$

According to equation (1), S can be expressed as:

$$S = \frac{E_{\mathrm{h}} - E_{\mathrm{c}}}{T_{\mathrm{h}} - T_{\mathrm{c}}} \tag{5}$$

where, E_{H} and E_{C} are the potentials at the hot and cold electrodes. By combining equations (3) and (5), S can be specified as:

$$S = \frac{\Delta E_{\mathrm{f}}}{\Delta T} + \frac{R}{nF\Delta T}\left[T_{\mathrm{h}}\ln\frac{[c_{\mathrm{A}}]_{\mathrm{h}}}{[c_{\mathrm{B}}]_{\mathrm{h}}} - T_{\mathrm{c}}\ln\frac{[c_{\mathrm{A}}]_{\mathrm{c}}}{[c_{\mathrm{B}}]_{\mathrm{c}}}\right] \tag{6}$$

For the pristine I_3^-/I^- ($I_3^- + 2e^- \rightarrow 3I^-$), the concentrations of redox species at the two sides are equal, namely, $[I_3^-]_h = [I_3^-]_c$, $[I^-]_h = [I^-]_c$ (subscript 'h' represents the hot side, 'c' represents thecold side). According to equation (6), the Seebeck coefficient (S) is expressed as:

$$S = \frac{\Delta E_{\mathrm{f}}}{\Delta T} = \frac{\Delta S_{\mathrm{re}}}{nF} \tag{7}$$

where, ΔS_{re} is the partial molar entropy.

Generally, the S of the pristine I_3^-/I^- is positive, equivalent to N-type.

When $[I_3^-]_h \neq [I_3^-]_c$, the corresponding S is calculated by equation (6) as follows:

$$S = \frac{\Delta E_{\mathrm{f}}}{\Delta T} + \frac{R}{2F\Delta T}\left[T_{\mathrm{h}} \ln \frac{\left[\mathrm{I}_3^-\right]_{\mathrm{h}}}{\left[\mathrm{I}^-\right]_{\mathrm{h}}^3} - T_{\mathrm{c}} \ln \frac{\left[\mathrm{I}_3^-\right]_{\mathrm{c}}}{\left[\mathrm{I}^-\right]_{\mathrm{c}}^3}\right] \tag{8}$$

If $[I_3^-]_h < [I_3^-]_c$, the second term on the right side of equation (8) will result in a negative concentration entropy. Thus, it is possible to reverse S from a positive value to a negative value.

When the temperature exceeds the phase transition temperature (T_p), the hydrogel becomes hydrophilic. Under a certain temperature difference (hot-side temperature (T_h) > T_p, cold-side temperature (T_c) < T_p), the hot side is hydrophilic (repelling I_3^-) and the cold side is hydrophobic (attracting I_3^-). The concentration of I_3^- at the hot side is lower than that at the cold side. When $[I_3^-]_h < [I_3^-]_c$, the S is a positive plus negative value. Therefore, the S may be negative, equivalent to P-type.

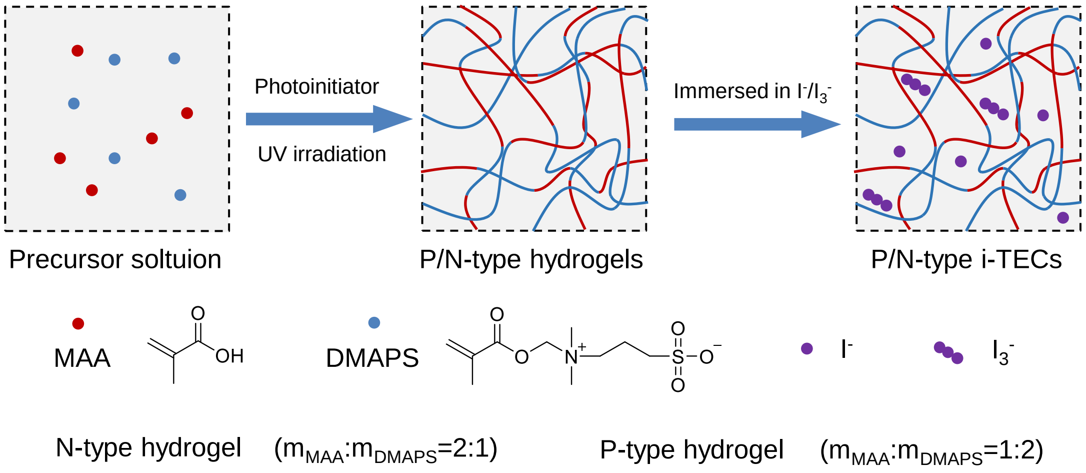

Fig. S1. Preparation of the N/P-type hydrogels and i-TECs.

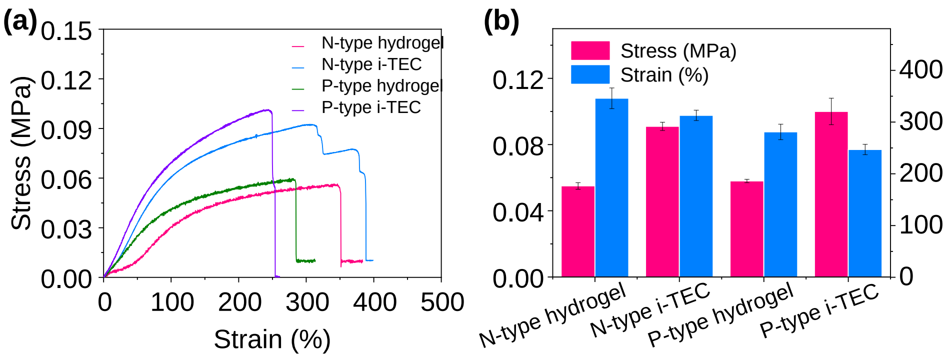

Fig. S2. (a) Stress-strain curve. (b) Maximum tensile strength and strain. The error bars were calculated using the standard deviation of the measured value.

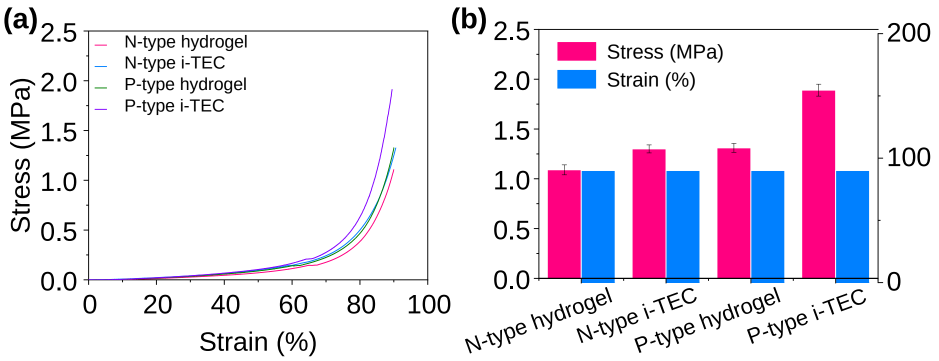

Fig. S3. (a) Compression curve. (b) Maximum compression strength and strain. The error bars were calculated using the standard deviation of the measured value.

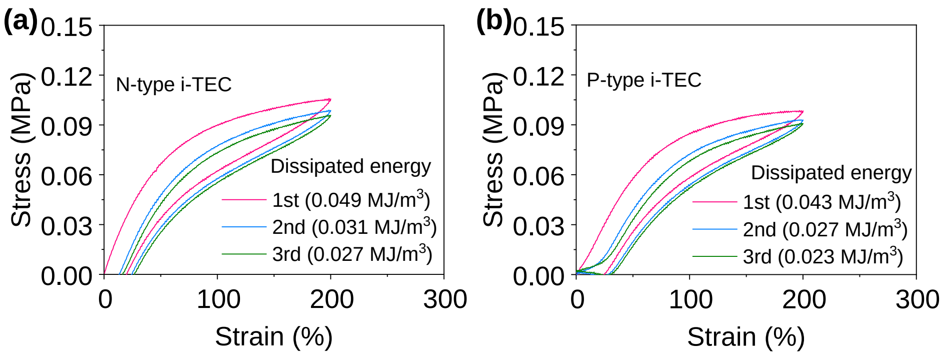

Fig. S4. Three consecutive tensile cycles with 200% strain. (a) N-type i-TEC. (b) P-type i-TEC.

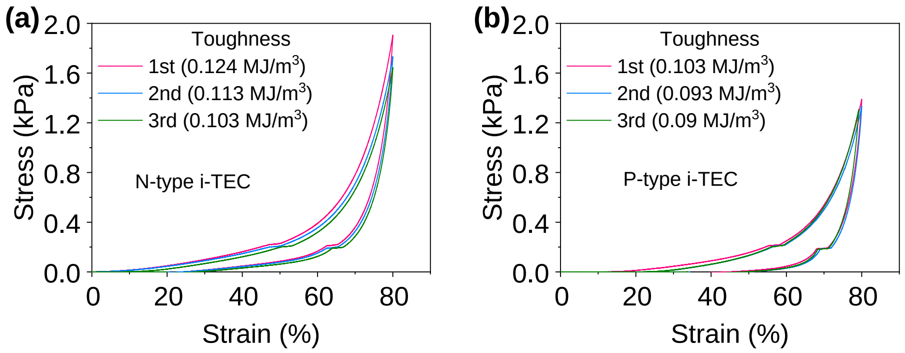

Fig. S5. Three consecutive compression cycles with 80% strain. (a) N-type i-TEC. (b) P-type i-TEC.

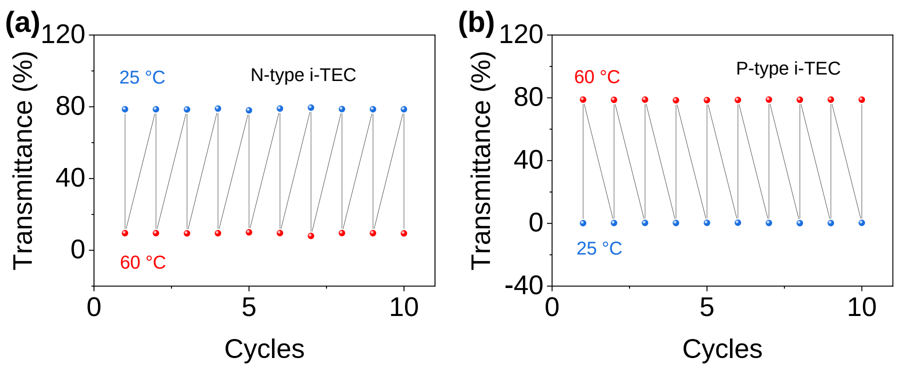

Fig. S6. Transmittance of i-TECs under cyclic heating and cooling at 25 ℃ and 60 ℃. (a) N-type i-TEC. (b) P-type i-TEC.

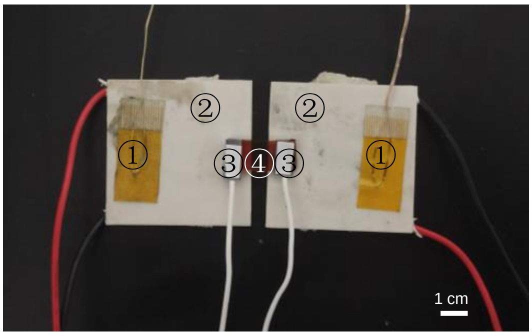

Fig. S7. Photo of the i-TEC test equipment. ① Thermocouple for real-time temperature recording. ② Peltier chip for constructing temperature difference. ③ Graphite paper electrode for interfacial redox reaction and electron transfer. ④ Hydrogel i-TEC with length, width and height of 20 mm, 10 mm and 3 mm. The measurement is carried out in a real environment (25 ℃, 75% relative humidity). To ensure the reliability of the measurement, we used 5mM I_3^-/I^- aqueous solution as a standard sample to calibrate the test device.

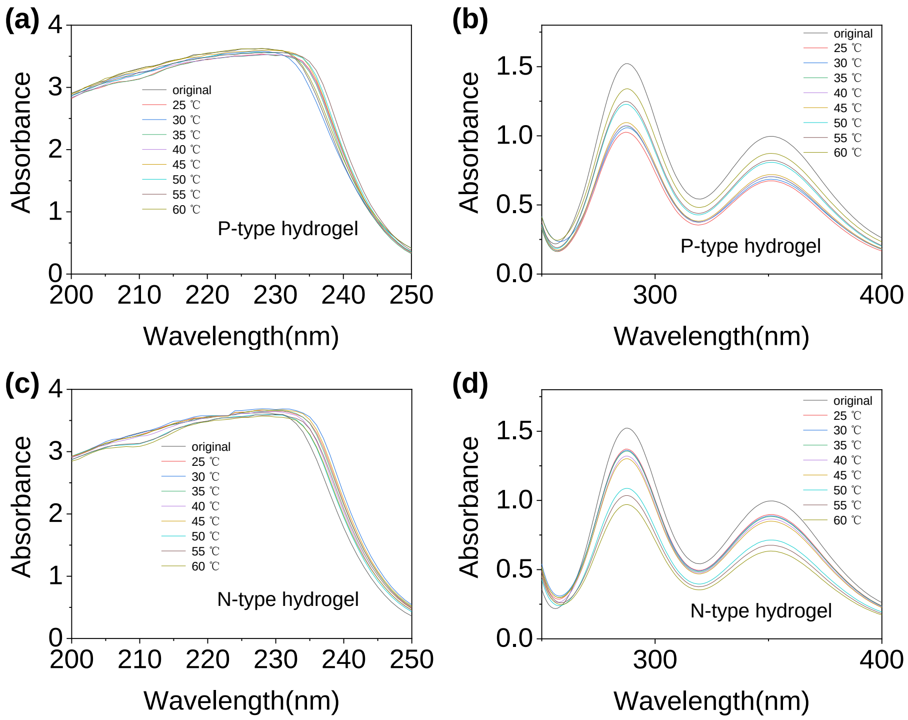

Fig. S8. UV–Vis spectra of the remaining solution after hydrogel soaking at different temperatures. (a,b) P-type hydrogel. (c,d) N-type hydrogel.

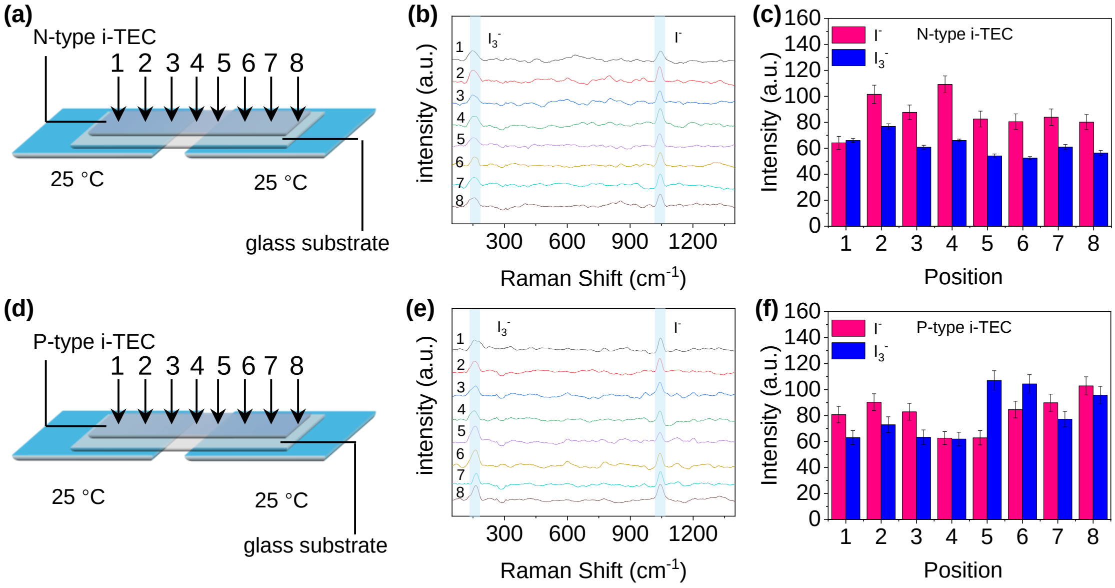

Fig. S9. (a) N-type i-TEC in-situ detection. (b) Raman spectra of the N-type i-TEC. (c) Intensity distribution of I_3^-/I^- in the N-type i-TEC. (d) P-type i-TEC in-situ detection. (e) Raman spectra of the P-type i-TEC. (f) Intensity distribution of I_3^-/I^- in the P-type i-TEC. The error bars were calculated using the standard deviation of the measured intensity.

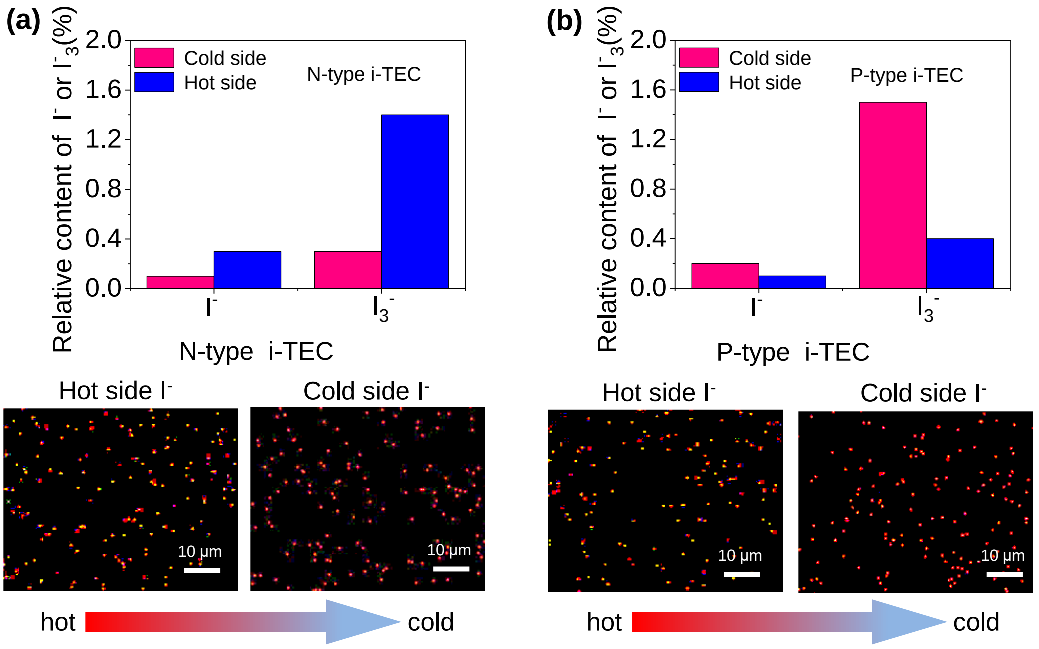

Fig. S10. Relative content of I_3^-/I^- and EDS mapping at hot side and cold side. (a) N-type i-TEC. (b) P-type i-TEC.

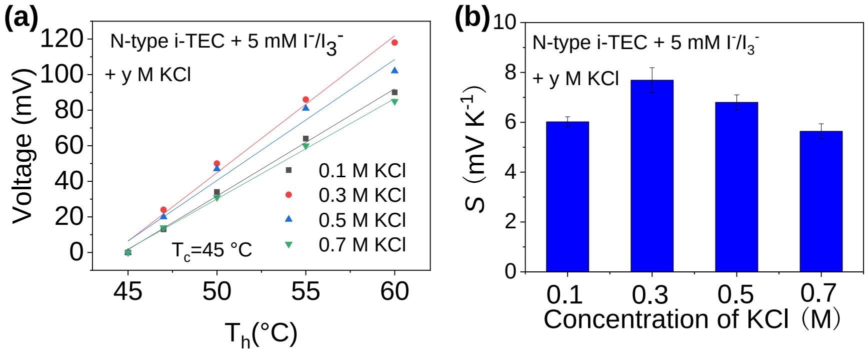

Fig. S11. (a) Measured open-circuit voltage (V_{oc}) of the N-type i-TEC versus the hot-side temperature. The cold-side temperature is maintained at 45 ℃. (b) Extracted seebeck coefficient. The error bars were calculated using the standard deviation of the measured seebeck coefficient.

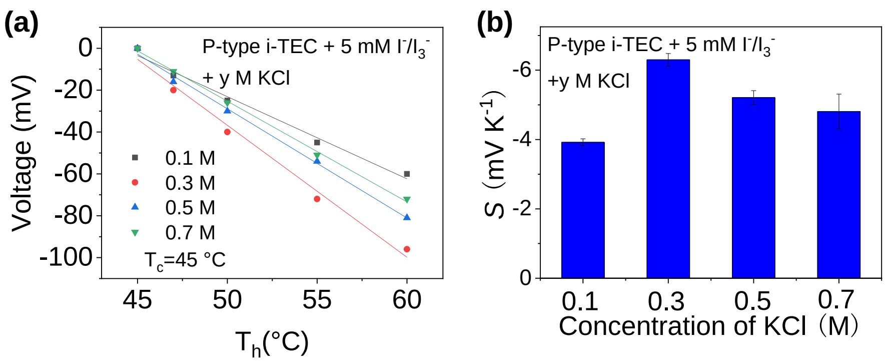

Fig. S12. (a) Measured open-circuit voltage (V_{oc}) of the P-type i-TEC versus the hot-side temperature. The cold-side temperature is maintained at 45 ℃. (b) Extracted seebeck coefficient. The error bars were calculated using the standard deviation of the measured seebeck coefficient.

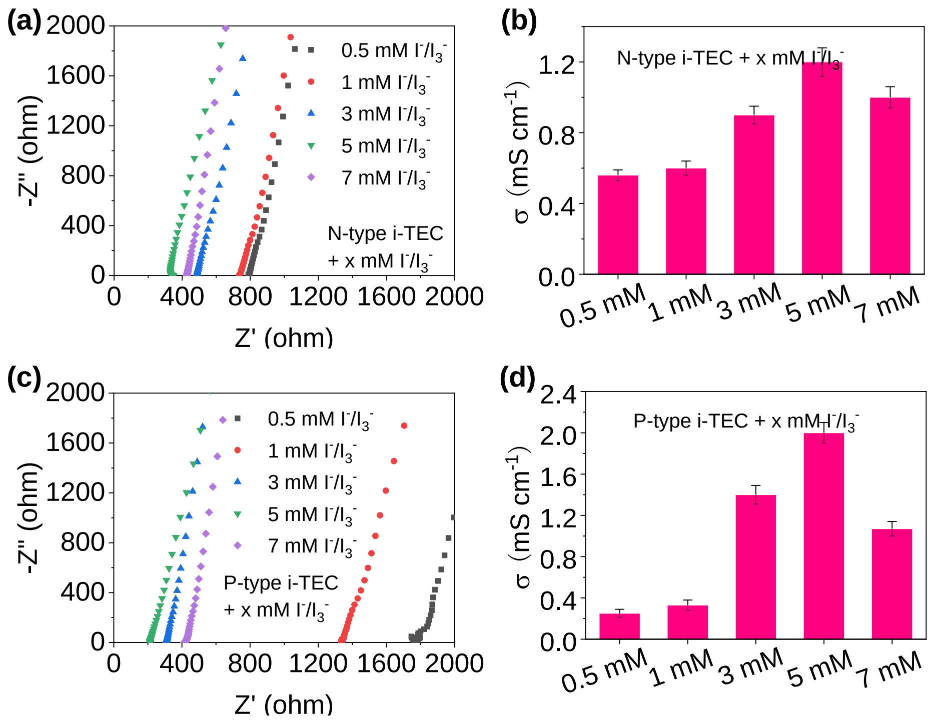

Fig. S13. Impedance curve and ionic conductivity of the i-TEC with different I_3^-/I^- contents. (a,b) N-type i-TEC without KCl addition. (c,d) P-type i-TEC without KCl addition. The error bars were calculated using the standard deviation of the measured ionic conductivity.

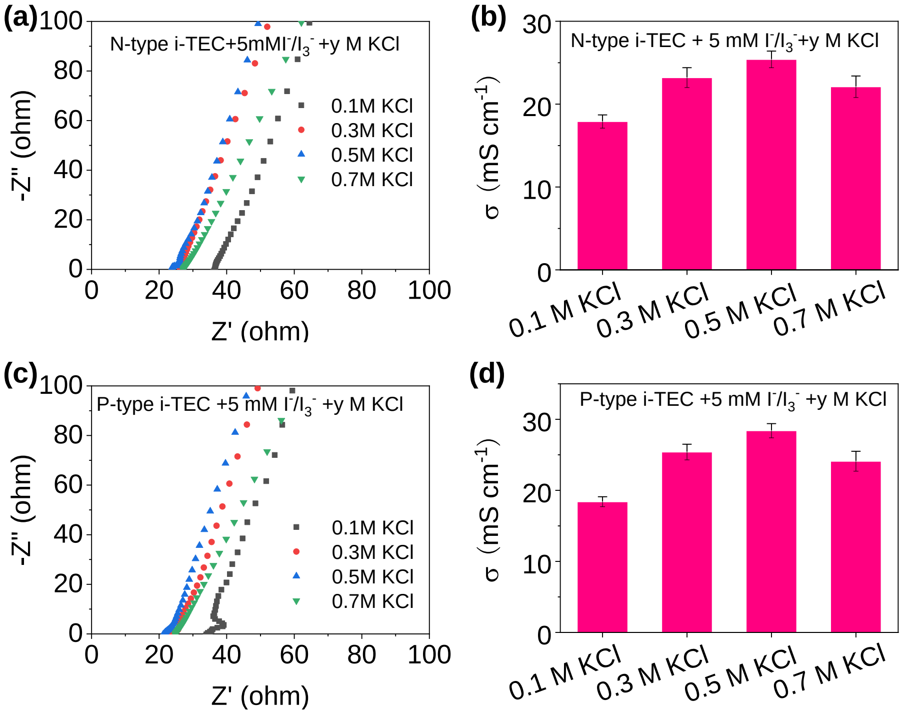

Fig. S14. Impedance curve and ionic conductivity of the i-TEC with different KCl contents. (a,b) N-type i-TEC. (c,d) P-type i-TEC. The error bars were calculated using the standard deviation of the measured ionic conductivity.

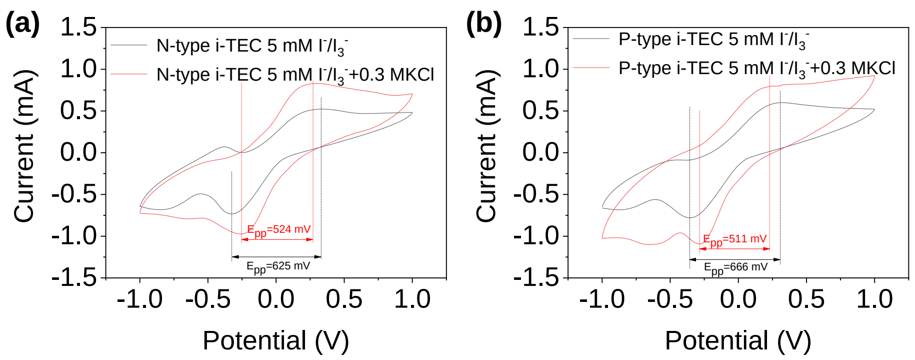

Fig. S15. (a) CV curve of the N-type i-TEC with/without KCl at 25 ℃. (b) CV curve of the P-type i-TEC with/without KCl at 25 ℃.

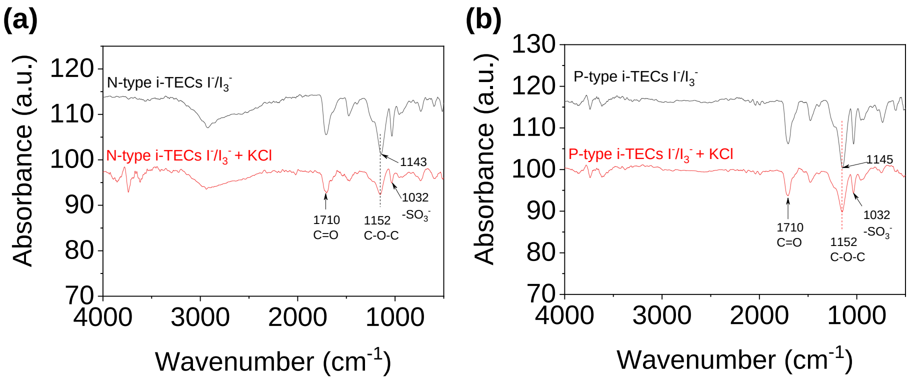

Fig. S16. (a) FTIR spectra of the N-type i-TEC with/without KCl. (b) FTIR spectra of the P-type i-TEC with/without KCl.

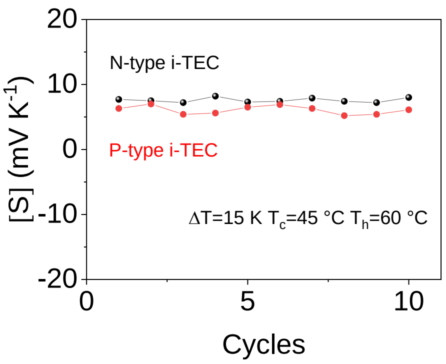

Fig. S17. Seebeck coefficient of i-TECs under cyclic establishing and removing temperature differences.

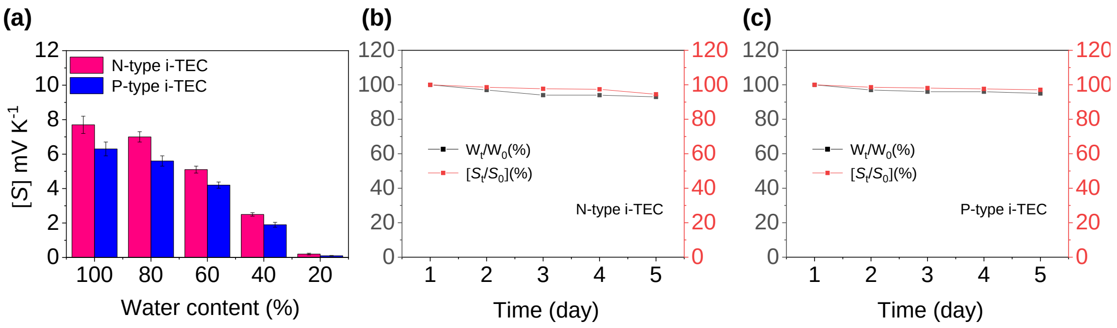

Fig. S18. (a) Effect of water content on the *S* of i-TECs. The error bars were calculated using the standard deviation of the *S*. (b) Change in water content and the *S* of the N-type i-TECs encapsulated with polyethylene film. (c) Change in water content and the *S* of the P-type i-TECs encapsulated with polyethylene film.

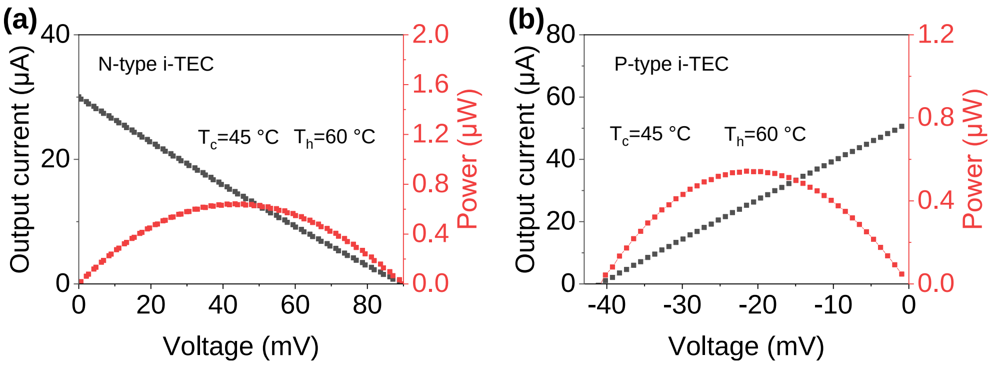

Fig. S19. Voltage-current-power. (a) N-type i-TEC without KCl addition. (b) P-type i-TEC without KCl addition.

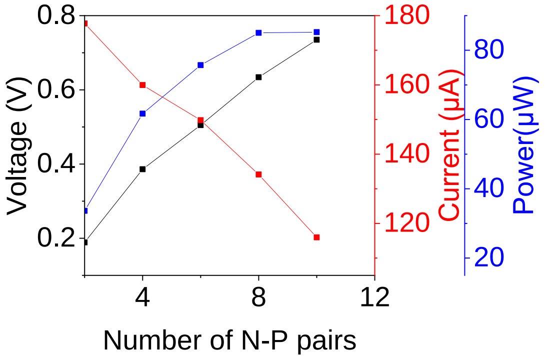

Fig. S20. Thermoelectric performance of multiple pairs of N-P i-TECs at $\Delta T = 35$ K.

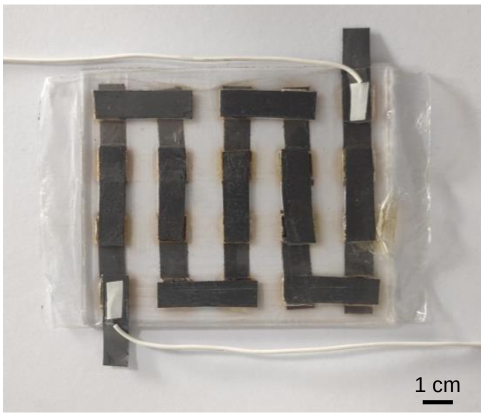

Fig. S21. Photo of the flexible thermoelectric cells manufactured by 10 pairs of N-P i-TECs.

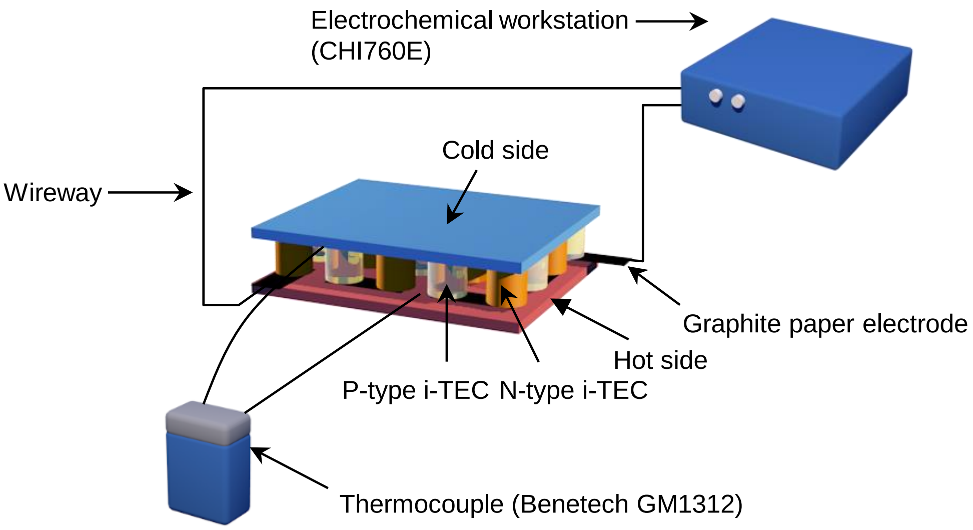

Fig. S22. Schematic image of a homemade Seebeck coefficient measurement device. The heating plate was located below the tested material to provide a temperature difference. The thermal voltage was recorded using an electrochemical workstation (CHI760E). The temperature at the hot and cold sides was recorded using a thermocouple (Benetech GM1312).

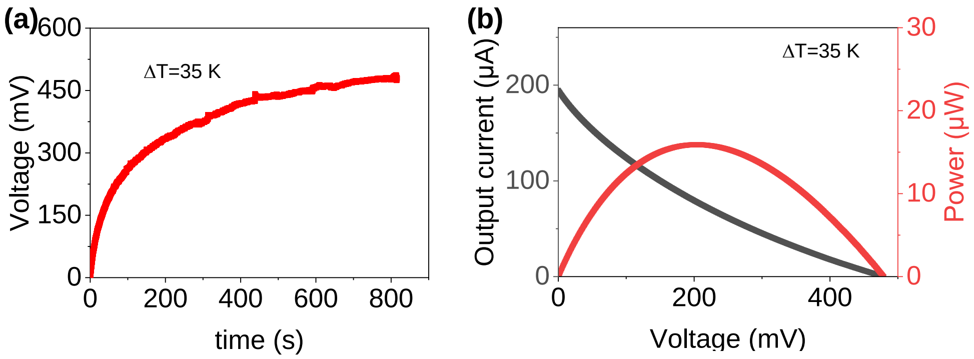

Fig. S23. (a) Output voltage and (b) power of 4 pairs of N-P i-TECs. The cold-side temperature is 25 ℃ and the hot-side temperature is 60 ℃.

Table S1. Comparison of thermoelectric performance between our i-TECs and the reported I_3^-/I^--based i-TECs

Letter	Electrolyte	*S* (mV K^{-1})	$P_{max}/\Delta T^2$ (mW m^{-2} K^{-2})	η_r (%)	Ref.
a	Phase transition hydrogel/KCl	7.7<br>-6.3	0.76<br>0.68	1<br>0.98	This work
b	[C_2mim] [BF_4] ionic liquid	-0.23<br>-0.39	0.0029<br>0.0017	0.00075<br>0.00025	[1]
c	PNIPAM nanogel/aqueous solution	1.91	0.07	--	[2]
d	KCl aqueous solution	-2	0.0147	0.033	[3]
e	Methyl-cellulose/KCl aqueous solution	-8.18<br>9.62	0.12<br>0.36	--<br>--	[4]
f	Dimethyl carbonate/ethylene carbonate solvent	7.7	0.21	--	[5]
g	CsCl aqueous solution	1.2	0.298	0.3	[6]
h	α-CD/KCl aqueous solution	2.4<br>4.2	--<br>--	0.009<br>0.028	[7]

Table S2. Comparison of thermoelectric performance of series integrated hydrogel i-TECs

**Electrolyte**	**VOC (V)**	P_{max} **(μW)**	**Cell number (n)**	**Ref.**
MAA/DMAPS/I_3^-/I^-/KCl	1.8	85	20	This work
I_3^-/I^-/PNIPAM nanogel	1	9	100	[2]
Gelatin-KCl-$FeCN^{3-/4-}$	2.2	5	25	[8]
PVA/$FeCN^{3-/4-}$	0.7	0.3	118	[9]
PVA/$Fe^{3+/2+}$/CMC/$FeCN^{3-/4-}$	0.34	39	36	[10]
PAAm/$Fe^{3+/2+}$/PAAm/$FeCN^{3-/4-}$	0.16	0.77	28	[11]
Gelatin-$FeCN^{3-/4-}$/Gr	0.12	4.2	20	[12]
Gelatin-KCl-$FeCN^{3-/4-}$	2.8	68	24	[13]

References:

1 Abraham, T. J., MacFarlane, D. R., Baughman, R. H., Jin, L. Y., Li, N. & Pringle, J. M. Towards ionic liquid-based thermoelectrochemical cells for the harvesting of thermal energy. *Electrochim. Acta* **113**, 87-93 (2013).

2 Duan, J. J., Yu, B. Y., Liu, K., Li, J., Yang, P. H., Xie, W. K., Xue, G. B., Liu, R., Wang, H. & Zhou, J. P-N conversion in thermogalvanic cells induced by thermo-sensitive nanogels for body heat harvesting. *Nano Energy* **57**, 473-479 (2019).

3 Zhou, H. Y., Yamada, T. & Kimizuka, N. Supramolecular thermo-electrochemical cells: enhanced thermoelectric performance by host-guest complexation and salt-induced crystallization. *J. Am. Chem. Soc.* **138**, 10502-10507 (2016).

4 Han, Y., Zhang, J., Hu, R. & Xu, D. Y. High-thermopower polarized electrolytes enabled by methylcellulose for low-grade heat harvesting. *Sci. Adv.* **8**, eabl5318 (2022).

5 Kim, K., Kang, J. & Lee, H. Hybrid thermoelectrochemical and concentration cells for harvesting low-grade waste heat. *Chem. Eng. J.* **426**, 131797 (2021).

6 Wang, H., Zhuang, X. Y., Xie, W. K., Jin, H. R., Liu, R., Yu, B. Y., Duan, J. J., Huang, L. & Zhou, J. Thermosensitive-CsI_3-crystal-driven high-power I^-/I_3^- thermocells. *Cell Rep. Phys. Sci.* **3**, 100737 (2022).

7 Liang, Y. M., Hui, J. K. H., Morikawa, M. A., Inoue, H., Yamada, T. & Kimizuka, N. High positive Seebeck coefficient of aqueous I^-/I_3^- thermocells based on host-guest interactions and LCST behavior of PEGylated α-cyclodextrin. *ACS Appl. Energ. Mater.* **4**, 5326-5331 (2021).

8 Han, C. G., Qian, X., Li, Q. K., Deng, B., Zhu, Y. B., Han, Z. J., Zhang, W. Q., Wang, W. C., Feng, S. P., Chen, G. & Liu, W. S. Giant thermopower of ionic gelatin near room temperature. *Science* **368**, 1091-1098 (2020).

9 Yang, P. H., Liu, K., Chen, Q., Mo, X. B., Zhou, Y. S., Li, S., Feng, G. & Zhou, J. Wearable thermocells based on gel electrolytes for the utilization of nody heat. *Angew. Chem. Int. Ed.* **55**, 12050-12053 (2016).

10 Liu, Y. Q., Zhang, S., Zhou, Y. T., Buckingham, M. A., Aldous, L., Sherrell, P. C., Wallace, G. G., Ryder, G., Faisal, S., Officer, D. L., Beirne, S. & Chen, J. Advanced wearable thermocells for body heat harvesting. *Adv. Energy Mater.* **10**, 2002539 (2020).

11 Xu, C., Sun, Y., Zhang, J. J., Xu, W. & Tian, H. Adaptable and wearable thermocell based on stretchable hydrogel for body heat harvesting. *Adv. Energy Mater.* **12**, 2201542 (2022).

12 Han, C. G., Zhu, Y. B., Yang, L. J., Chen, J. W., Liu, S. J., Wang, H. Y., Ma, Y. M., Han, D. X. & Niu, L. Remarkable high-temperature ionic thermoelectric performance induced by graphene in gel thermocells. *Energy Environ. Sci.* **17**, 1559-1569 (2024).

13 Li, Y. C., Li, Q. K., Zhang, X. B., Deng, B., Han, C. G. & Liu, W. S. 3D hierarchical electrodes boosting ultrahigh power output for gelatin-KCl-$FeCN^{4-}/^{3-}$ ionic thermoelectric cells. *Adv. Energy Mater.* **12**, 2103666 (2022).
